# Risk prediction in pulmonary hypertension due to chronic heart failure: incremental prognostic value of pulmonary hemodynamics

**DOI:** 10.1186/s12872-022-02492-1

**Published:** 2022-02-16

**Authors:** Ruilin Quan, Shian Huang, Lingpin Pang, Jieyan Shen, Weifeng Wu, Fangming Tang, Xiulong Zhu, Weiqing Su, Jingzhi Sun, Zaixin Yu, Lemin Wang, Xianyang Zhu, Changming Xiong, Jianguo He

**Affiliations:** 1 Center of Pulmonary Vascular Disease, State Key Laboratory of Cardiovascular Disease, Fuwai Hospital, National Center for Cardiovascular Diseases, Chinese Academy of Medical Sciences and Peking Union Medical College, No. 167, Beilishi Road, Xicheng District, Beijing, 100037 China; 2 Cardiovascular Medicine Center, Affiliated Hospital of Guangdong Medical University, Zhanjiang, China; 3 Department of Cardiology, Renji Hospital, School of Medicine, Shanghai Jiao Tong University, Shanghai, China; 4 Department of Cardiology, First Affiliated Hospital of Guangxi Medical University, Nanning, China; 5 Department of Cardiology, Nongken Central Hospital of Guangdong Province, Zhanjiang, China; 6 Department of Cardiovascular Medicine, The People’s Hospital of Gaozhou, Maoming, China; 7 Department of Cardiovascular Medicine, The People’s Hospital of Lianjiang, Zhanjiang, China; 8 Department of Cardiolody, Affiliated Hospital of Jining Medical University, Jining, China; 9 Department of Cardiology, Xiangya Hospital of Central South University, Changsha, China; 10 Department of Cardiology, Tongji Hospital Affiliated To Tongji University, Shanghai, China; 11 Department of Congenital Heart Disease, General Hospital of Shenyang Military Area Command, Shenyang, China

**Keywords:** Pulmonary hypertension, Heart failure, Pulmonary hemodynamics, Survival, Risk prediction

## Abstract

**Background:**

There is no generally accepted comprehensive risk prediction model cooperating risk factors associated with heart failure and pulmonary hemodynamics for patients with pulmonary hypertension due to left heart disease (PH-LHD). We aimed to explore outcome correlates and evaluate incremental prognostic value of pulmonary hemodynamics for risk prediction in PH-LHD.

**Methods:**

Consecutive patients with chronic heart failure undergoing right heart catheterization were prospectively enrolled. The primary endpoint was all-cause mortality. Individual variable selection was performed by machine learning methods. Cox proportional hazards models were conducted to identify the association between variables and mortality. Incremental value of hemodynamics was evaluated based on the Seattle heart failure model (SHFM) and Meta-Analysis Global Group in Chronic Heart Failure (MAGGIC) scores.

**Results:**

A total of 276 PH-LHD patients were enrolled, with a median follow-up time of 34.7 months. By L1-penalized regression model and random forest approach, diastolic pressure gradient (DPG) and mixed venous oxygen saturation (SvO_2_) were the hemodynamic predictors most strongly associated with mortality (coefficient: 0.0255 and -0.0176, respectively), with consistent significance after adjusted for SHFM [DPG: HR 1.067, 95% CI 1.024–1.113, *P* = 0.022; SvO_2_: HR 0.969, 95% CI 0.953–0.985, *P* = 0.002] or MAGGIC (DPG: HR 1.069, 95% CI 1.026–1.114, *P* = 0.011; SvO_2_: HR 0.970, 95% CI 0.954–0.986, *P* = 0.004) scores. The inclusion of DPG and SvO_2_ improved risk prediction compared with using SHFM [net classification improvement (NRI): 0.468 (0.161–0.752); integrated discriminatory index (IDI): 0.092 (0.035–0.171); likelihood ratio test: *P* < 0.001] or MAGGIC [NRI: 0.298 (0.106–0.615); IDI: 0.084 (0.033–0.151); likelihood ratio: *P* < 0.001] scores alone.

**Conclusion:**

In PH-LHD, pulmonary hemodynamics can provide incremental prognostic value for risk prediction.

*Clinical trial registration*: NCT02164526 at https://clinicaltrials.gov.

## Introduction

Pulmonary hypertension due to left heart disease (PH-LHD), typically characterized by a passive increase in pulmonary artery wedge pressure (PAWP) in response to a backward transmission of elevated left-sided filling pressures, is the most frequent cause of pulmonary hypertension (PH) [1, 2]. PH-LHD can result from a variety of etiology, but it is most common in left ventricular systolic or diastolic dysfunction, which is also referred to as heart failure with reduced or preserved ejection fraction (HFrEF or HFpEF) [3, 4].Independent of the left ventricular ejection fraction (LVEF) and stage of heart failure (HF), PH is associated with increased hospitalization and mortality [5]. As the important prognostic value of PH-LHD has been increasingly identified in HF patients, several studies have explored and reported potential risk predictors associated with the outcomes in PH-LHD [5–10]. Of those, several pulmonary hemodynamic variables obtained from right heart catheterization (RHC), which is the gold standard to diagnose PH, have been reported to be prognostic predictors for survival and/or other outcomes, such as transpulmonary pressure gradient (TPG), diastolic pressure gradient (DPG) and pulmonary vascular pressure (PVR) [5–10]. However, their prognostic value is still controversial, which can be attributed to several reasons, such as the diverse study designs, the heterogeneous study cohorts, and the various analyzed covariates [5–10]. As regards HF, which is one of the most common background etiologies of PH-LHD, in addition to hemodynamic variables, other HF-associated risk factors have also been reported to have prognostic value, such as age, blood pressure, and the level of serum creatinine [6, 8]. Therefore, a comprehensive risk prediction model, which cooperates demographics, clinical assessments, comorbid conditions, biomarkers, hemodynamics and other functional tests, is essential to the risk stratification for patients with PH due to HF, and it can further help clinicians identify patients at high risk, guide treatment strategies, and improve clinical management.

However, to the date, the most commonly applied comprehensive risk prediction strategies for PH, including the REVEAL risk score (RRS), the risk assessment proposed by the 2015 European Society of Cardiology (ESC)/European Respiratory Society (ERS) guidelines and its abbreviated versions, have been derived from pulmonary arterial hypertension (PAH), while its application to PH-LHD has yet to be validated [4, 11–14]. On the other hand, as regards the generally accepted risk stratification methods for HF, such as the Seattle heart failure model (SHFM) and Meta-Analysis Global Group in Chronic Heart Failure (MAGGIC) scores, hemodynamic variables of pulmonary circulation are not included, which means when simply applying those methods to patients with PH-LHD, potential prognostic information provided by hemodynamics could be ignored [15, 16]. Agarwal R et al. have reported a prognostic tool in PH-HFpEF, which incorporates clinical and hemodynamic measures [17]. However, several important hemodynamic variables like pulmonary arterial compliance (PAC) and DPG, which later have been reported to have prognostic value and even indicate potential involvement of structural remodeling in PH-LHD, have not been included in the study [6–9, 17].

Accordingly, the objectives of our study were: (1) to evaluate prognostic value of the established risk prediction strategies for PAH and HF in a cohort of PH-LHD and identify other outcome correlates; (2) to explore incremental prognostic value of pulmonary hemodynamics for risk prediction in PH-LHD.

## Methods

### Patient enrollment

In this national multicenter prospective registry study, patients with symptomatic chronic heart failure undergoing first right heart catheterization (RHC) during hospitalization were consecutively enrolled from January 2013 to August 2016 in 11 participating medical centers throughout China. The study has been registered on ClinicalTrials.gov (Identifier: NCT02164526). Patients in the current study were included if: (1) their age were between 18 and 80; (2) they were diagnosed with chronic heart failure with NYHA II-IV; (3) the etiology of their heart failure was ischemic heart disease, hypertension or idiopathic cardiomyopathy; (4) they underwent RHC and had hemodynamically defined post-capillary PH. Patients were excluded if they: (1) had pulmonary hypertension of other types; (2) had organic valvular heart disease; (3) had right ventricular outflow tract obstruction; (4) had pericardial disease, including pericarditis, a thickened or calcified pericardium; (5) had chronic pulmonary diseases such as interstitial fibrosis, chronic obstructive pulmonary disease or primary parenchymal lung disease; (6) had hypertrophic obstructive cardiomyopathy; and (7) previous acute myocardial infarction and/or received revascularization in the previous six months**.** Written informed consent was obtained from all enrolled patients. This study complied with the Declaration of Helsinki. The study protocol was approved by the ethics committee of Fuwai hospital (Approval No. 2012-401).

### Measurements and data collection

Electrocardiogram (ECG), chest X ray, transthoracic echocardiography, pulmonary function test, high-resolution computed tomography of the chest, ventilation/perfusion scintigraphy lung scan (if necessary) and pulmonary angiography (if necessary) were performed to evaluate the severity of heart failure, exclude pulmonary hypertension due to other causes and conditions in the exclusion criteria, such as lung diseases or chronic thromboembolism pulmonary hypertension.

The indications of RHC were based upon referring physicians, mostly for the diagnosis of suspected pulmonary hypertension with an elevated systolic pulmonary arterial systolic pressure (PASP) at echocardiography, for decreased exercise capacity despite initial standard therapies, or for assessment and monitoring of hemodynamics. Catheterizations were also performed for transplantation eligibility assessments, or prior to percutaneous interventions or surgical procedures. Catheterizations were performed in stable, non-acute clinical conditions; in other words, patients with acute decompensated HF were excluded, with only stable chronic HF patients enrolled.

For patients who met the inclusion and exclusion criteria, the following data were collected: (1) demographics, medical history, clinical symptoms and vital signs; (2) results of laboratory tests, echocardiography and catheterizations; and (3) drug treatments (patients were optimally managed by HF treatments, with no usage of PH-targeted drugs).

### RHC

RHC was centralized using a standard protocol across all the participating centers. During the procedure, patients were positioned supine with legs flat. A 7F Swan-Ganz catheter (Edwards Lifesciences World Trade Co. Ltd, Irvine, CA, USA) was inserted from a femoral or jugular approach, with zeroing calibration in the mid-axillary line to atmospheric pressure. Correct positioning of the catheter was verified by chest fluoroscopy. Hemodynamics, including right atrial pressure (RAP), diastolic pulmonary arterial pressure (dPAP), systolic pulmonary arterial pressure (sPAP), mean pulmonary arterial pressure (mPAP) and PAWP, were obtained during spontaneous breathing. All pressures were recorded after at least 6 stable cardiac cycles during several respiratory cycles. PAWP was measured at end-diastole. Cardiac output (CO) was measured by the thermodilution method (the mean value of three-time measurements). The DPG was assessed as the difference between dPAP and PAWP. TPG was calculated by subtracting PAWP from mPAP. PVR was obtained by the equation: PVR = (mPAP-PAWP)/CO. PAC was calculated as stroke volume divided by the difference between sPAP and dPAP.

### Definitions

Symptomatic chronic heart failure was diagnosed based on the presence of HF signs, symptoms and other indications as recommended in the 2012 HF guidelines, which differentiates HFpEF from HFrEF by echocardiographically estimated LVEF, with a cut-off value of 50% [3]. Post-capillary pulmonary hypertension, also referred to as PH-LHD, was hemodynamically defined as mPAP ≥ 25 mmHg and PAWP > 15 mmHg [4]. The cutoff values of hemodynamic variables were determined in terms of PH guidelines’ recommendations [4].

As for PH risk prediction strategies, the algorithm incorporated 9 evaluable elements used to calculate RRS: NYHA functional class II, renal insufficiency, males whose age > 60 years, systolic blood pressure (SBP), heart rate (HR), N-terminal pro-brain natriuretic peptide (NT-proBNP), RAP and PVR [11, 18]. Data for WHO Group I subgroup, 6-min walk distance (6MWD) and diffusing capacity of the lung for carbon monoxide (DLCO) were not available for the current cohort and were therefore omitted; however, the RRS only requires 7/12 evaluable elements to maintain significant predictive power and calibration [18].

The French registry invasive risk stratification method classifies patients according to the presence of the ESC/ERS low-risk criteria [14]. As NT-proBNP and mixed venous oxygen saturation (SvO_2_) were available but 6MWD was not available in the current cohort, we counted the number of the following low-risk criteria: (1) NYHA functional class II, (2) RAP < 8 mmHg, (3) cardiac index ≥ 2.5 L·min^−1^·m^−2^, (4) NT-proBNP < 300 ng/L and (5) SvO_2_ > 65%.

As regards the risk prediction scores for heart failure, SHFM and MAGGIC risk scores were calculated in the current study as previous reported [15, 16]. As some variables for the SHFM model were missing in the current registry (white blood cell, lymphocytes and diuretic daily dose), the following variables were used to create a slightly revised version of the SHFM: age, gender, body mass index (BMI), NYHA class, SBP, ischemic etiology, LVEF, hemoglobin, sodium, creatinine, angiotensin converting enzyme inhibitor use or angiotensin receptor blocker use, beta blocker use, digoxin use, diuretic use (daily dose not available), statin use, allopurinol use, the usage of devices. All variables included in the MAGGIC method were available and included in the calculation [15].

### Primary endpoint and follow-up

The primary endpoint of this study was all-cause mortality. Overall survival was measured from the date of RHC to the date of death from any cause. Follow-up was performed by telephone calls, outpatient visits or inpatient admissions every 6 months ± 2 weeks. In each follow-up, it was confirmed whether patients died or received transplantations and whether they had received any surgical or intervention treatments or had any instances of cardiac hospitalization. Patients were followed until death or until the end of the study (July 2017).

### Statistical analysis

Continuous variables are presented as the mean ± standard deviations, or median (interquartile range [IQR]). For normally distributed variables, the differences between groups were compared by the Student’s t test, while for non-normally distributed variables, the analyses were carried out by the Mann–Whitney test. Categorical variables are shown as frequencies and percentages, and chi-square tests were used to compare the differences between two subgroups. The performance of two established risk prediction strategies for PAH (i.e., RRS and the French registry invasive risk stratification method) and two risk scores for HF (i.e., the SHFM and MAGGIC risk scores) were evaluated by Harrell’s C-index in the current cohort.

To identify variables associated with mortality, L1-penalized regression model (LASSO) with tenfold cross-validation and the random forest approach were applied. Based on previous literature and clinical relevance, the following variables were assessed: demographics (age, sex, and smoke status), clinical assessments (BMI, blood pressure, and NYHA functional class), comorbidities (arterial hypertension, coronary heart disease, diabetes mellitus and cardiomyopathy), clinically assessed biomarkers [NT-proBNP, hemoglobin (Hb), creatinine, blood urea nitrogen (BUN) and sodium], echocardiographic and hemodynamic parameters, as well as treatment strategies. As the variables selected by both methods were included either in the SHFM or in the MAGGIC risk scores, and also due to the reason that the sample size as well as the number of events of the study cohort were relatively small, the analyses regarding hemodynamics thereafter were based on the two HF risk scores.

Cox proportional hazards analyses were performed to compute hazard ratios (HRs) with 95% confidence intervals (CIs). The proportional hazards assumption was examined by inspection of Schoenfeld residuals. In bivariate Cox analyses, which included hemodynamic variables and SHFM or MAGGIC scores, multiple testing was adjusted by Bonferroni correction. Only hemodynamic variables, which were selected by both the LASSO and random forest approach, and remained consistently significant after adjusted by SHFM or MAGGIC scores in the bivariate Cox analyses, were considered to be included in the HF scores to evaluate their incremental prognostic value. Model performance was evaluated for discrimination and calibration. Discrimination power was assessed by Harrell’s C-index and compared by net reclassification improvement (NRI) and integrated discriminatory index (IDI), while likelihood ratio test and Akaike information criteria (AIC) were used to assess calibration properties. Internal validation was estimated with bootstrapping. Differences were considered statistically significant when the two-sided *P* value was < 0.05. All analyses were performed with R statistical package (version 4.0.0, R Foundation for Statistical Computing, Vienna, Austria).

## Results

### Study cohort

**Fig. 1:**
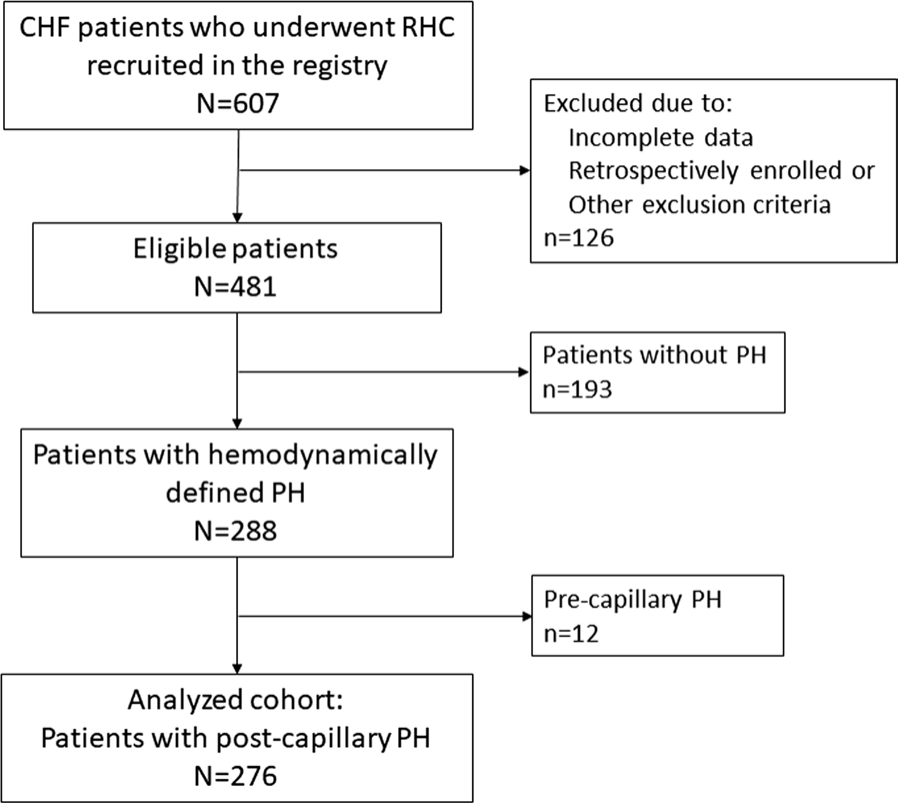
Patient dispositions. CHF: chronic heart failure; RHC: right heart catheterization; PH: pulmonary hypertension

A total of 607 patients who underwent RHC were recruited in the registry. Of those, 276 post-capillary PH patients who met the inclusion and exclusion criteria were enrolled in the current study (Fig. 1). The mean age of the cohort was 63.2 ± 12.4 years, and the majority of the patients were males (71.7%). 79 (28.6%) patients were classified as HFrEF, while 197 (71.4%) patients were diagnosed as HFpEF. The most common comorbid condition was coronary artery disease (73.6%), while arterial hypertension, hyperlipidemia and diabetes accounted for 35.9%, 27.9% and 24.6% of the overall cohort, respectively. A mean LVEF of 53.9 ± 10.8% was identified by echocardiography, and the median NT-proBNP was 2580 fmol/L (IQR 1017 to 4289). As regards hemodynamic parameters obtained by RHC, the mean level for mPAP was 31.7 ± 7.62 mmHg and 5.67 ± 4.91 mmHg for DPG. Baseline characteristics are shown in Table 1.

**Table 1:** Baseline characteristics of the study cohort

Variables	Overall cohort (N = 276)	Survivors (n = 223)	Non-survivors (n = 53)	*P* value*
Age (years)	62 ± 12	62 ± 12	68 ± 12	**0.001**
Male, n (%)	198 (71.7)	156 (70.0)	42 (79.2)	0.177
Body mass index (kg/m^2^)	22.97 ± 2.83	22.92 ± 2.97	23.19 ± 2.12	0.251
Heart rate (beats/min)	77 ± 15	77 ± 15	77 ± 15	0.794
Systolic blood pressure (mmHg)	131.68 ± 21.86	131.01 ± 20.64	134.51 ± 26.44	0.752
Diastolic blood pressure (mmHg)	75.65 ± 11.75	75.96 ± 11.94	74.36 ± 10.96	0.269
**NYHA functional class, n (%)**				**0.004**
III	79 (28.6)	62 (27.8)	17 (32.1)	
IV	20 (7.2)	9 (4.0)	11 (20.8)	
**Medical history**				
Current smoker, n (%)	68 (24.6)	51 (22.9)	17 (32.1)	0.372
Coronary artery disease, n (%)	203 (73.6)	161 (72.2)	42 (79.2)	0.296
Arterial hypertension, n (%)	99 (35.9)	75 (33.6)	24 (45.3)	0.112
Cardiomyopathy, n (%)	41 (14.9)	35 (15.7)	6 (11.3)	0.421
Atrial fibrillation, n (%)	8 (2.9)	6 (2.7)	2 (3.8)	0.673
Diabetes, n (%)	68 (24.6)	55 (247)	13 (24.5)	0.984
Hyperlipidemia, n (%)	77 (27.9)	61 (27.4)	16 (30.2)	0.679
Cerebrovascular disease, n (%)	19 (6.9)	15 (6.7)	4 (7.5)	0.832
Renal insufficiency, n (%)	18 (6.5)	12 (5.4)	6 (11.3)	**< 0.001**
Liver insufficiency, n (%)	3 (1.1)	2 (3.8)	1 (0.4)	**0.036**
Thyroid disorders, n (%)	7 (2.5)	6 (2.7)	1 (1.9)	0.738
**Laboratory tests**				
Red blood cell count	4.51 ± 0.75	4.58 ± 0.72	4.21 ± 0.82	**0.001**
Platelet count	217.64 ± 66.16	218.71 ± 68.5	213.15 ± 55.6	0.795
Platelet distribution width	15.48 ± 2.95	15.36 ± 2.31	15.99 ± 4.79	**0.039**
Erythrocyte sedimentation rate	39.25 ± 72.23	37.9 ± 75.86	44.94 ± 54.61	**0.010**
Hematocrit (%)	37.81 ± 10.09	37.66 ± 9.39	38.45 ± 12.71	0.233
Hemoglobin (g/L)	132.23 ± 19.91	133.86 ± 19.01	125.39 ± 22.25	**0.003**
Alanine aminotransferase (IU/L)	34.22 ± 51.23	32.42 ± 33.75	41.8 ± 94.59	0.069
Aspartate aminotransferase (IU/L)	42.39 ± 103.99	36.83 ± 48.29	65.81 ± 215.74	0.502
Total bilirubin (μmol/L)	13.67 ± 8.52	13.84 ± 8.84	12.93 ± 7.07	0.608
Albumin (g/L)	40.05 ± 14.57	40.52 ± 16.04	38.09 ± 4.41	**0.037**
Sodium (mmol/L)	139.88 ± 4.08	139.92 ± 4.15	139.73 ± 3.79	0.617
Creatinine (μmol/L)	86.39 ± 39.65	81.75 ± 26.56	105.88 ± 69.46	**0.044**
Blood urea nitrogen (mmol/L)	5.8 ± 2.68	5.56 ± 2.31	6.8 ± 3.74	**0.026**
Uric acid (mmol/L)	373.96 ± 123.19	362.14 ± 117.87	423.67 ± 133.52	**0.007**
Triglyceride (mmol/L)	1.93 ± 2.15	1.9 ± 2.05	2.05 ± 2.52	0.619
Cholesterol (mmol/L)	6.92 ± 34.13	7.42 ± 37.95	4.78 ± 2.46	0.159
HDL-C (mmol/L)	1.17 ± 0.37	1.19 ± 0.37	1.09 ± 0.35	0.063
LDL-C (mmol/L)	2.75 ± 1.22	2.79 ± 1.24	2.61 ± 1.12	0.280
Glucose (mmol/L)	6.22 ± 3.52	6.04 ± 2.69	6.94 ± 5.82	0.833
NT-proBNP (fmol/mL) ^#^	2580 (1017, 4289)	2381.0 (838.6, 4249.0)	3698.0 (1421.0, 6271.5)	**0.015**
hsCRP	30.06 ± 38.16	27.84 ± 35.23	39.37 ± 47.91	0.195
**Echocardiography**				
Left atrial anteroposterior diameter (mm)	35.86 ± 6.83	35.75 ± 6.72	36.3 ± 7.32	0.431
Left ventricular end diastolic diameter (mm)	49.99 ± 8.26	49.7 ± 8.05	51.17 ± 9.06	0.205
Right ventricular anteroposterior diameter (mm)	19.93 ± 5.72	20.05 ± 5.62	19.45 ± 6.13	0.953
Left ventricular ejection fraction (%)	53.88 ± 10.78	54.05 ± 11.01	53.19 ± 9.82	0.319
Pericardial effusion, n (%)	12 (4.3)	10 (4.5)	2 (3.8)	0.820
**Hemodynamics**				
mRAP (mmHg)	14.32 ± 4.4	14.16 ± 4.32	15.02 ± 4.72	0.127
RVSP (mmHg)	46.69 ± 12.92	45.66 ± 12.12	51 ± 15.24	**0.011**
RVEDP (mmHg)	14.21 ± 5.77	13.93 ± 5.88	15.36 ± 5.18	**0.025**
sPAP (mmHg)	46.56 ± 11.51	45.52 ± 10.84	50.94 ± 13.23	**0.002**
dPAP (mmHg)	23.35 ± 6.44	22.82 ± 6.37	25.57 ± 6.31	**0.002**
mPAP (mmHg)	31.68 ± 7.62	31.05 ± 7.38	34.36 ± 8.09	**0.001**
PAWP (mmHg) ^#^	16 (16, 18)	16 (16, 19)	16 (16, 16)	0.703
DPG (mmHg)	5.67 ± 4.91	5.2 ± 4.69	7.66 ± 5.34	**0.001**
PVR (WU)	5.89 ± 4.45	5.68 ± 4.45	6.77 ± 4.38	0.063
Cardiac index (L/min/m^2^)	2.47 ± 0.78	2.44 ± 0.76	2.63 ± 0.82	0.083
SvO_2_ (%)	70.81 ± 19.33	73 ± 19.3	61.61 ± 16.69	** < 0.001**
PAC (mL/mmHg)	4.88 ± 4.36	5.17 ± 4.55	3.67 ± 3.17	**0.038**
**Treatments**				
ACEI, n (%)	124 (44.9)	97 (43.5)	27 (50.9)	0.327
ARB, n (%)	59 (21.4)	45 (20.2)	14 (26.4)	0.320
Beta blocker, n (%)	185 (67.0)	146 (65.5)	39 (73.6)	0.259
Ca^2+^ channel blockers, n (%)	51 (18.5)	35 (15.7)	16 (30.2)	**0.015**
Diuretics, n (%)	135 (48.9)	102 (45.7)	33 (62.3)	**0.031**
Digoxin, n (%)	63 (22.8)	46 (20.6)	17 (32.1)	0.074
MRA, n (%)	153 (55.4)	115 (51.6)	38 (71.7)	**0.008**
Statin, n (%)	238 (86.2)	190 (85.2)	48 (90.6)	0.308
Antiplatelet/anticoagulant, n (%)	57 (20.7)	50 (22.4)	7 (13.2)	0.136

*P* values < 0.05 shown in bold

NYHA, New York Heart Association; HDL-C, high-density lipoprotein cholesterol; LDL-C, low-density lipoprotein cholesterol; NTpro-BNP, N-terminal pro b-type natriuretic peptide; hsCRP, high sensitivity C reactive protein; RAP, right atrial pressure; RVSP, right ventricular systolic pressure; RVEDP, right ventricular end diastolic pressure; sPAP, systolic pulmonary artery pressure; dPAP, diastolic pulmonary artery pressure; mPAP, mean pulmonary artery pressure; PAWP, pulmonary arterial wedge pressure; DPG, diastolic pressure gradient; PVR, pulmonary vascular resistance; SvO_2_, mixed venous oxygen saturation; PAC, pulmonary arterial compliance; ACEI, angiotensin-converting enzyme inhibitors; ARB, angiotensin receptor blocker; MRA, mineralocorticoid receptor antagonist

*Comparison between survivors and non-survivors

^#^Median (interquartile range)

### Survival

During a median follow-up time of 34.7 months (IQR 17.77 to 39.63), 53 patients died. The 3- year survival estimate of the study cohort was 79.3% (95% CI: 74.3–84.8%). In addition, 4 patients underwent pacemaker implantation, while no cardiac resynchronization therapy, implantable cardioverter defibrillator implantation, radiofrequency ablation, or any other interventions/operations were carried out.

As shown in Table 1, compared to survivors, patients who died were older (68.36 ± 11.64 versus 62.00 ± 12.32 years, *P* = 0.001), had worse NYHA class (*P* = 0.004) and higher NT-proBNP [3698.0 (IQR 1421.0 to 6271.5) versus 2381.0 (838.6 to 4249.0) fmol/L, *P* = 0.015], more frequently had CKD (11.3 versus 5.4%, *P* < 0.001), with worse hemodynamics, as indicated by higher mPAP (34.36 ± 8.09 versus 31.05 ± 7.38 mmHg, *P* = 0.001), DPG (7.66 ± 5.34 versus 5.20 ± 4.69 mmHg, *P* = 0.001), RVSP (51.0 ± 15.24 versus 45.66 ± 12.12 mmHg, *P* = 0.011), RVEDP (15.36 ± 5.18 versus 13.93 ± 5.88 mmHg, *P* = 0.025) and lower SvO_2_ (61.61 ± 16.69 versus 73.0 ± 19.3%, *P* < 0.001).

### Prognostic value of the established risk strategies

The median of the SHFM score was 2 (range from 0 to 3), and the mean of the MAGGIC score was 18 ± 6 points. Both strategies showed significant prognostic value in the univariate Cox models, and could discriminate survivors from non-survivors, indicated by a C-index of 0.593 (95% CI: 0.519–0.667) and 0.62 (0.540–0.700), respectively (Table 2).

**Table 2:** Prognostic value of HF and PH risk stratification strategies for all-cause mortality

Variables	HR (95% CI)	*P* value	Statistic (95% CI)	*P* value for Likelihood ratio test
**Risk stratification strategies for HF**				
SHFM (HR for per point increase)	1.639 (1.140–2.356)	0.008	0.593 (0.519–0.667)	0.01
MAGGIC (HR for per 5 points increase)	1.490 (1.224–1.815)	< 0.001	0.62 (0.540–0.700)	< 0.001
**Risk stratification strategies for PH**				
REVEAL score (HR for per point increase)	1.293 (1.097–1.523)	0.002	0.634 (0.552–0.716)	0.002
French abbreviated method (HR for per criteria increase)	0.744 (0.562–0.986)	0.040	0.587 (0.505–0.669)	0.04

HF: heart failure; PH: pulmonary hypertension; HR: hazard ratio; CI: confidence interval; SHFM: the Seattle heart failure model; MAGGIC: Meta-Analysis Global Group in Chronic Heart Failure

We also applied the established PH risk stratification strategies–the REVEAL risk score and the French registry invasive method–to the study cohort. The median level for the REVEAL score was 8 (range from 4 to 13), while the majority of the patients met two or one low risk criteria included in the French method, which accounted for 34.8% and 31.9% of the analyzed cohort, respectively. The two PH risk prediction strategies demonstrated C-statistic of 0.634 (0.552–0.716) and 0.587 (0.505–0.669), respectively (Table 2).

### Outcome correlates

**Fig. 2:**
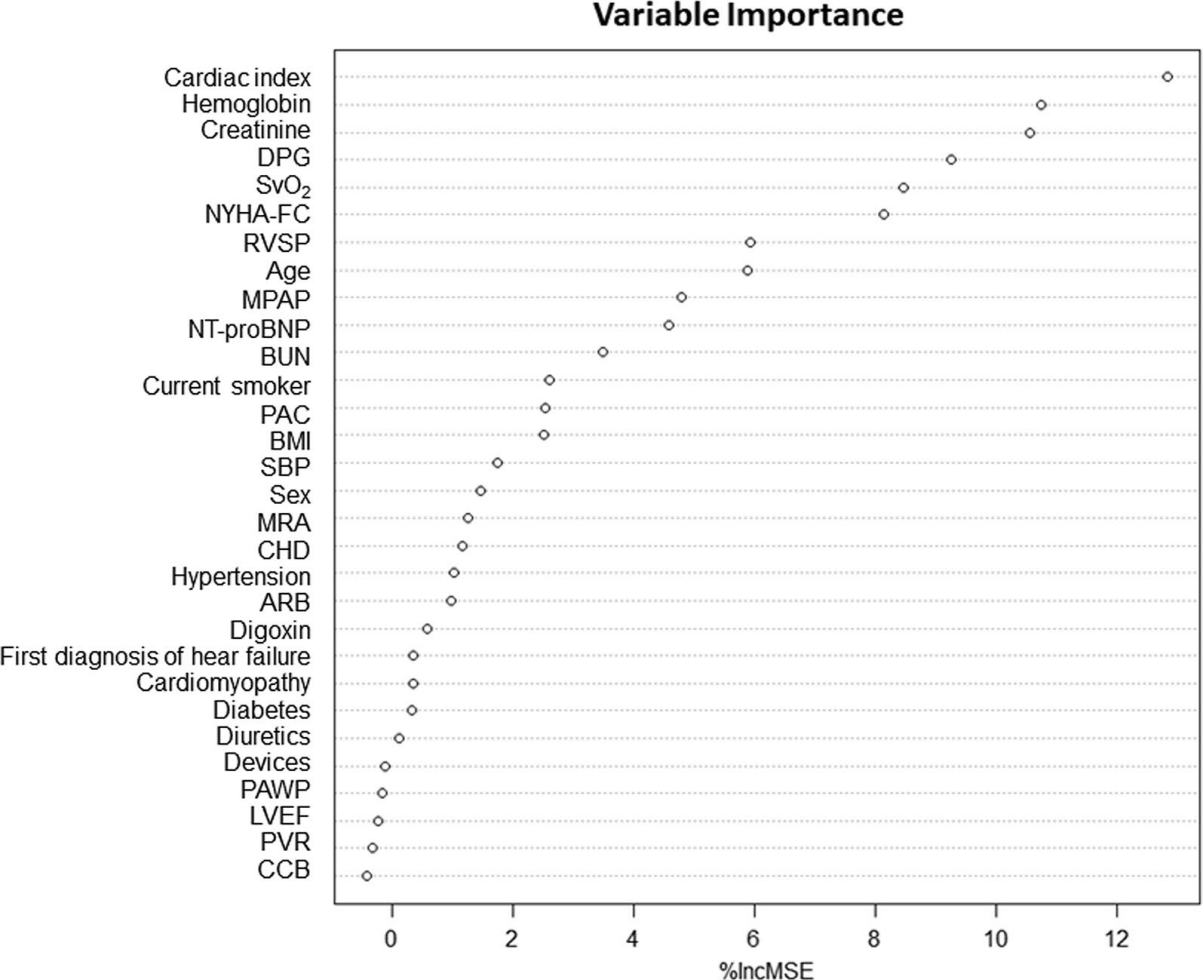
The variable importance plot based on the random forest classifier with the indicator of “all-cause mortality” as the dependent variable. DPG: diastolic pressure gradient; SvO_2_: mixed venous oxygen saturation; NYHA-FC: New York Heart Association functional class; RVSP: right ventricular systolic pressure; MPAP: mean pulmonary artery pressure; NTpro-BNP: N-terminal pro b-type natriuretic peptide; BUN: blood urea nitrogen; PAC: pulmonary arterial compliance; BMI: body mass index; SBP: systolic blood pressure; MRA: mineralocorticoid receptor antagonist; CHD: coronary heart disease; ARB: angiotensin-receptor blockers; PAWP: pulmonary arterial wedge pressure; LVEF: left ventricular ejection fraction; PVR: pulmonary vascular resistance; CCB: Ca2 + channel blockers

The LASSO analysis extracted the following 10 predictors most robustly related to all-cause mortality: age, NYHA functional class, Hb, creatinine, RVSP, DPG, PAC, CI, SvO_2_, and the use of angiotensin-converting enzyme inhibitor. The coefficients of the above predictors were shown in Table 3. As regards the random forest approach, the 10 top-scoring predictors were cardiac index, Hb, creatinine, DPG, SvO_2_, NYHA functional class, RVSP, age, mPAP, and NT-proBNP (Fig. 2). As indicated, in addition to the hemodynamic parameters associated with pulmonary circulation, variables that reflected the status of HF were also importantly associated with outcome. Hence, to analyze those HF-related predictors together with RHC-measured hemodynamics, SHFM and MAGGIC risk score were used thereafter to avoid overfitting. In terms of the results of both variable selection approaches, RVSP, DPG, SvO_2_ and CI were identified as the most discriminative hemodynamic variables.

**Table 3:** Estimates of coefficients for the predictors of all-cause mortality with nonzero coefficients in the L1-penalized regression model (LASSO)

Variables	Coefficient
Age	0.0055
NYHA functional class	0.5223
Hemoglobin	− 0.0007
Creatinine	0.0029
RVSP	0.0018
DPG	0.0255
PAC	− 0.0049
CI	0.2885
SvO_2_	− 0.0176
ACEI	− 0.1023

RVSP, right ventricular systolic pressure; DPG, diastolic pressure gradient; PAC, pulmonary arterial compliance; CI, cardiac index; SvO2, mixed venous oxygen saturation; ACEI, angiotensin-converting enzyme inhibitor

### Univariate and bivariate Cox analyses

As shown in Table 4, after adjusted by SHFM risk score, only DPG (HR 1.067, 95% CI 1.024–1.113, *P* = 0.022) and SvO_2_ (HR 0.969, 95% CI 0.953–0.985, *P* = 0.002) remained to be significant predictors for all-cause mortality. When adjusted by MAGGIC score, dPAP (HR 1.281, 95% CI 1.097–1.495, *P* = 0.022), DPG (HR 1.069, 95% CI 1.026–1.114, *P* = 0.011), CI (HR 1.792, 95% CI 1.228–2.615, *P* = 0.022) and SvO_2_ (HR 0.970, 95% CI 0.954–0.986, *P* = 0.004) were independent predictors for mortality. Thus, DPG and SvO_2_ were selected to be included in the SHFM or MAGGIC scores thereafter to identify their incremental prognostic value.

**Table 4:** Univariate and bivariate Cox hazard proportional analyses of hemodynamics for all-cause mortality

Variables	Unadjusted HR (95% CI)	*P* value	SHFM-adjusted HR (95% CI)	*P* value*	MAGGIC-adjusted HR (95% CI)	*P* value*
mRAP (per 1 mmHg increase)	1.045 (0.981–1.113)	0.200	1.046 (0.982–1.114)	1	1.057 (0.989–1.129)	1
RVSP (per 10 mmHg increase)	1.280 (1.069–1.531)	**0.007**	1.280 (1.067–1.535-)	0.096	1.273 (1.062–1.527)	0.108
RVEDP (per 5 mmHg increase)	1.127 (0.935–1.359)	0.209	1.113 (0.925–1.340)	1	1.137 (0.935–1.382)	1
sPAP (per 10 mmHg increase)	1.313 (1.091–1.581)	**0.004**	1.299 (1.075–1.570)	0.084	1.279 (1.057–1.548)	0.144
dPAP (per 5 mmHg increase)	1.232 (1.059–1.435)	**0.007**	1.230 (1.056–1.433)	0.96	1.281 (1.097–1.495)	**0.024**
mPAP (per 5 mmHg increase)	1.189 (1.048–1.349)	**0.007**	1.187 (1.042–1.353)	0.120	1.203 (1.058–1.367)	0.060
PAWP (per 1 mmHg increase)	0.985 (0.927–1.045)	0.609	0.989 (0.934–1.046)	1	0.998 (0.941–1.059)	1
DPG (per 1 mmHg increase)	1.066 (1.027–1.106)	**< 0.001**	1.067 (1.024–1.113)	**0.024**	1.069 (1.026–1.114)	**0.012**
PVR (per 1 WU increase)	1.037 (0.983–1.093)	0.189	1.045 (0.989–1.104)	1	1.040 (0.985–1.098)	1
Cardiac index (per 1 L/min/m^2^ increase)	1.317 (0.938–1.849)	0.112	1.466 (1.030–2.088)	0.408	1.792 (1.228–2.615)	**0.024**
PAC (per mL/mmHg increase)	0.928 (0.856–1.006)	0.068	0.920 (0.848–0.998)	0.540	0.937 (0.865–1.014)	1
SvO_2_ (per 5% increase)	0.857 (0.789–0.913)	**< 0.001**	0.969 (0.953–0.985)	**0.002**	0.970 (0.954–0.986)	**0.004**

*P* values < 0.05 shown in bold

HR, hazard ratio; CI, confidence interval; SHFM, the Seattle heart failure model; MAGGIC, Meta-Analysis Global Group in Chronic Heart Failure; mRAP, mean right atrial pressure; RVSP, right ventricle systolic pressure; RVEDP, right ventricular end diastolic pressure; sPAP, systolic pulmonary artery pressure; dPAP, diastolic pulmonary artery pressure; mPAP, mean pulmonary artery pressure; PAWP, pulmonary arterial wedge pressure; DPG, diastolic pressure gradient; PVR, pulmonary vascular resistance; PAC, pulmonary arterial compliance; SvO_2_, mixed venous oxygen saturation

*P value after Bonferroni correction

### Incremental prognostic value of hemodynamics

**Fig. 3:**
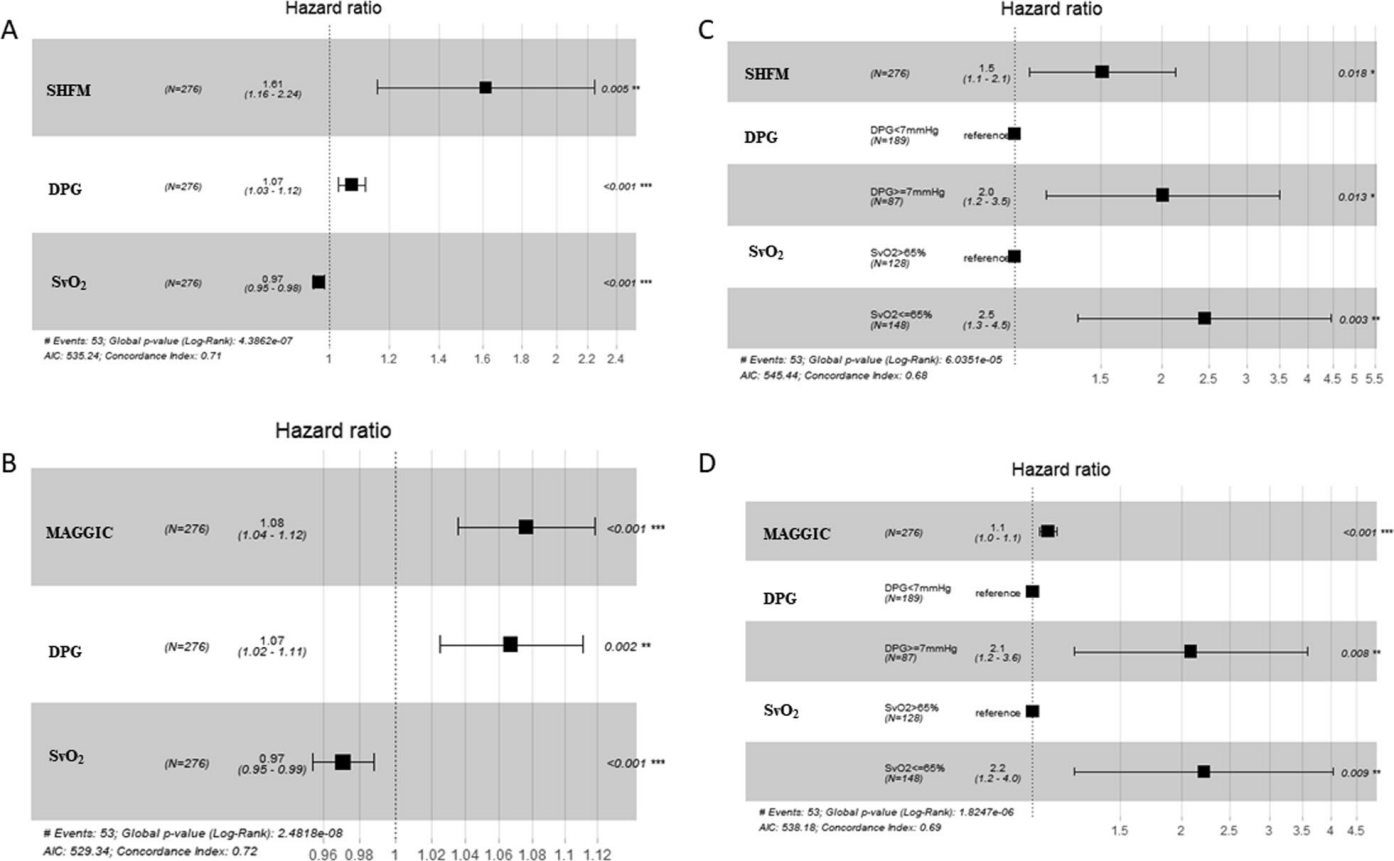
Multivariate Cox hazard proportional models for all-cause mortality. **A** SHFM + hemodynamics as continuous variables; **B** MAGGIC + hemodynamics as continuous variables; **C** SHFM + hemodynamics as dichotomous variables; **D** MAGGIC + hemodynamics as dichotomous variables. DPG: diastolic pressure gradient; SvO_2_: mixed venous oxygen saturation; SHFM: the Seattle heart failure model; MAGGIC: Meta-Analysis Global Group in Chronic Heart Failure

We established two multivariate Cox models by adding DPG and SvO_2_ to the SHFM or the MAGGIC score respectively (Fig. 3A, B). Table 5 shows that the inclusion of DPG and SvO_2_ improved risk prediction compared with the models only involving the SHFM [NRI: 0.468 (0.161–0.752); IDI: 0.092 (0.035–0.171); LR test: *P* < 0.001] or MAGGIC [NRI: 0.298 (0.106–0.615); IDI: 0.084 (0.033–0.151); LR test: *P* < 0.001] scores.

**Table 5:** Comparison of risk prediction models before and after the addition of hemodynamics for all-cause mortality

	C-statistic	AIC	LR test, p value	NRI (95% CI)	P value	IDI (95% CI)	P value
**SHFM**							
SHFM only	0.593 (0.519–0.667)	557	–	–	–	–	–
SHFM + hemodynamics^a^ (continuous)^c^	0.715 (0.648–0.782)	535	< 0.001	0.468 (0.161–0.752)	< 0.01	0.092 (0.035–0.171)	< 0.01
SHFM + hemodynamics^a^ (dichotomous^b^)^c^	0.684 (0.610–0.758)	545	< 0.001	0.230 (0.029–0.642)	< 0.01	0.064 (0.018–0.141)	< 0.01
**MAGGIC**							
MAGGIC only	0.620 (0.540–0.700)	549	–	–	–	–	–
MAGGIC + hemodynamics^a^ (continuous)^d^	0.716 (0.647–0.785)	529	< 0.001	0.298 (0.106–0.615)	0.02	0.084 (0.033–0.151)	< 0.01
MAGGIC + hemodynamics^a^ (dichotomous^b^)^d^	0.689 (0.613–0.765)	538	< 0.001	0.162 (0.016–0.510)	0.01	0.066 (0.028–0.155)	< 0.01

AIC, Akaike information criteria; LR test, likelihood ratio test; NRI, net reclassification improvement; IDI, integrated discriminatory index; CI, confidence interval; SHFM, the Seattle heart failure model; MAGGIC, Meta-Analysis Global Group in Chronic Heart Failure

^a^Hemodynamics included: diastolic pressure gradient (DPG) and mixed venous oxygen saturation (SvO_2_)

^b^Cutoff values for DPG and SvO_2_: 7 mmHg and 65%, respectively

^c^Compared to SHFM risk score

^d^Compared to MAGGIC risk score

In addition, DPG and SvO2 were further added to the HF scores as dichotomous variables using the guidelines recommended cutoff values of 7 mmHg and 65%, respectively (Fig. 3C, D), and the two variables also provided incremental prognostic value beyond the traditional HF risk scores [for SHFM: NRI: 0.230 (0.029–0.642), IDI: 0.064 (0.018–0.141), LR test: *P* < 0.001; for MAGGIC: NRI: 0.162 (0.016–0.510), IDI: 0.066 (0.028–0.155), LR test: *P* < 0.001] (Table 5).

## Discussion

The present study has validated the performance of four established PH or HF risk prediction strategies in patients with PH-LHD, and has identified a broad range of variables, including demographics, clinical assessments, biomarkers and hemodynamics, associated with all-cause mortality of PH-LHD. We extracted DPG and SvO_2_ as the most robustly significant hemodynamic variables, and after their addition to the SHFM or MAGGIC scores, the model performance for predicting mortality improved significantly.

Neither the PH risk assessment strategies nor the HF risk scores demonstrated satisfactory performance for discriminating survivors from non-survivors in the current PH-LHD cohort. As regards the two modalities for PH, both methods ignore risk factors associated with HF nature to some extent, such as the medications for HF treatment, variables reflecting the function of left-sided heart, and even the etiologies of HF like ischemic heart disease. Notably, among the REVEAL score and the French method, the former tool showed a better discrimination power (AIC: 554 versus 559, *P* < 0.001), which could somehow be explained by the reason that compared to the French method, the REVEAL score involved additional risk factors related to demographics and comorbid conditions [14, 18]. Thus, it emphasizes the importance of those variables for risk prediction in PH-LHD. On the other hand, in HF patients, the SHFM and MAGGIC risk scores have been widely used for outcome prediction [15, 16]. However, the assessment for hemodynamics of pulmonary circulation is not included in those HF scores, though several hemodynamic variables like mPAP, DPG, PVR and PAC have been reported to have critical prognostic value in patients with HF or PH-LHD [6–8]. Hence, the lack of hemodynamic parameters in the two scores could partly explain the relatively poor model performance in the current cohort. As the current study focused on the patients who were complicated with PH, changes of pulmonary hemodynamics cannot be ignored. However, previous studies evaluating the performance of the two HF risk scores did restrict their cohort in patients with PH, which could explain the discrepancy of the performance of the two scores between our cohort and previous literature. As the HF background should not be omitted in patients with PH-LHD, we further added hemodynamics on the basis of the SHFM and MAGGIC risk scores, and significant improvements of model performance for predicting mortality have been illustrated.

Due to the relatively small sample size and the number of events available, instead of traditional univariate and multivariate Cox analyses, we utilized machine learning methods to select potential prognostic predictors, which were also further validated by Cox models. By LASSO and random forest approach, a range of data, including demographics, exercise capacity, biomarkers and hemodynamics, were found to be associated with the outcomes of PH-LHD, which verifies the critical need for a comprehensive risk prediction model to perform stratification. After strict variable selection, among hemodynamic parameters, only DPG and SvO_2_ were involved in the analysis thereafter. Other hemodynamic variables were not included because of either losing significance after adjustments or not showing the most robust significance for risk prediction, which could be attributed to the interaction and association with other factors.

With the hemodynamic profiling of PH-LHD being constantly evolving, PH-LHD is further classified into two subtypes, based on the involvement of pre-capillary components: isolated post-capillary PH (Ipc-PH) with pure increasing pressure in post-capillary components, and combined post- and pre-capillary PH (Cpc-PH) with potential pulmonary vascular remodeling [1, 2]. The two distinct hemodynamic phenotypes have been defined in terms of several parameters obtained by RHC, such as TPG, DPG and PVR [1, 2, 19]. However, the optimal definition is still under debate because none of the variables are free from limitations regarding not only the different sensitivity of distinguishing Cpc-PH from Ipc-PH, but the inconsistent prognostic value in diverse study samples of PH-LHD [19]. In the current study, after strict variable selection, DPG illustrated consistent significance for outcome prediction, which could somehow imply its further usage for prediction in PH-LHD, and its prognostic value might also be regarded as an indicator of pre-capillary involvement in HF patients [2]. In the current study, after adjustment, SvO_2_ also remained to be a significant predictor for outcomes in PH-LHD. SvO_2_ is an important hemodynamic parameter derived from RHC, reflecting the balance between O_2_ delivery and consumption [20]. A decrease in SvO_2_ indicates a cardiac output insufficiency to meet systemic requirements, which is a critical physiological change in HF [20, 21]. Furthermore, it is one of the most robust hemodynamic indicators of right ventricular function and prognosis, and has been widely used in the risk stratification of PH [4]. Although recently there have been research exploring novel noninvasive estimation of SvO_2_ by echocardiography and expired gas analysis, as guidelines stated, noninvasive assessment has not yet been sufficiently validated to allow routine clinical use [4, 22].

In addition to systolic or diastolic dysfunction of left ventricle, other types of left heart disease can also cause pulmonary hypertension with potential different characteristics and mechanism from heart failure. Unlike heart failure induced by coronary artery disease, cardiomyopathy or arterial hypertension, where the changes in pulmonary circulation are caused mainly by systolic or diastolic dysfunction of left ventricle, other types of left heart disease, such as valvular heart disease, often affect pulmonary hemodynamics by their inherent mechanical dysfunction. Thus, in those scenarios, though they can also cause heart failure and pulmonary hypertension, the changes in pulmonary hemodynamics can be attributed to two aspects: heart failure and mechanical changes. In the current study, as we tried to identify the characteristics of pulmonary hypertension induced by heart failure, we excluded those with comorbidities that could have potential effects on pulmonary hemodynamics due to other mechanical reasons in addition to systolic or diastolic dysfunction of left ventricle.

The majority of patients enrolled in the current study have less advanced heart failure, indicated by relatively better NYHA FC, LVEF or mPAP. Actually, instead of patients with severe HF hospitalized in Cardiac Care Unit (CCU) or Heart Failure Care Unit (HFCU), patients enrolled in the current study admitted to hospital mainly due to requirements for standard follow-ups of heart failure, with/without decreased exercise capacity, or for further examinations of the underlying etiologies of heart failure, such as coronary artery disease. Usually, due to the invasive nature of RHC, patients with coronary heart disease, for whom the coronary angiography is required in their follow-ups, are more willing to receive RHC, while in other etiologies, a great percentage of patients are more likely to decline to receive this invasive examination despite the need for assessment of hemodynamics. It should be noted that patients with coronary heart disease in out cohort underwent RHC in combination with coronary angiography and left heart catheterization. Therefore, this could explain the high percentage of patients with coronary artery disease enrolled in the study. In addition, it should be noted that, in our country, most invasive examinations, such as coronary angiography, left and right heart catheterizations, are required to be conducted during hospitalization, though most patients are in a stable condition. Thus, the majority of patients underwent RHCs during their follow-up hospitalizations for chronic heart failure**.**

Our study has several limitations. First, there could be an inherent patient selection bias in our study due to the relatively small sample size. The limited sample size could be attributed to our inclusion criteria, as we only enrolled patients undergoing RHC, which was not common in clinical practice among patients with left heart disease. It should be noted that although we found incremental prognostic value of hemodynamic variables in risk prediction, it not necessarily requires all HF patients to receive RHC in daily practice. Instead, it implies that among patients who undergo RHC due to suspected PH or other indications, an incorporation of hemodynamics and traditional risk prediction strategies for HF could better help stratification. Inherent patient selection bias could also attribute to the less advanced HF patients enrolled in the study, as mentioned above. Another main limitation is the small number of events, which could affect the number of variables entering the Cox regression analyses. Accordingly, to avoid overfitting, we applied machine learning algorithms to select the risk factors most strongly associated with mortality, and on the other hand, the SHFM and MAGGIC scores, which integrated HF-related risk factors, were utilized as composite variables to enter Cox analyses. In addition, except the MAGGIC score, the other three risk prediction models explored in the current study all have some variables not available, which could potentially affect the model performance. Notably, the lack of data on diuretics dose is one of the potential limitations of the current study. The SHFM score included dose of diuretics as one of the prognostic variables; however, in a study reported by Bilchick et al., where diuretic use was also not available, the authors created a slightly revised version of the SHFM score with comparable statistical power [23]. Nevertheless, it also indicates that in real-world clinical practice, the variables included in the reported risk scores cannot always be all collected in different conditions, which emphasizes the importance of maximumly taking advantage of data available to help risk prediction.

## Conclusion

In PH-LHD, pulmonary hemodynamics can provide incremental prognostic value for risk prediction.

## Data Availability

The dataset analyzed in the current study are available from the corresponding author on reasonable request.

## References

[1] Naeije R, Gerges M, Vachiery JL, Caravita S, Gerges C, Lang IM (2017). Hemodynamic phenotyping of pulmonary hypertension in left heart failure. Circ Heart Fail.

[2] Guazzi M, Naeije R (2017). Pulmonary hypertension in heart failure: pathophysiology, pathobiology, and emerging clinical perspectives. J Am Coll Cardiol.

[3] McMurray JJ, Adamopoulos S, Anker SD, Auricchio A, Bohm M, Dickstein K, Falk V, Filippatos G, Fonseca C, Gomez-Sanchez MA et al. ESC guidelines for the diagnosis and treatment of acute and chronic heart failure 2012: The Task Force for the Diagnosis and Treatment of Acute and Chronic Heart Failure 2012 of the European Society of Cardiology. Developed in collaboration with the Heart Failure Association (HFA) of the ESC. Eur J Heart Fail 2012, 14(8):803–869. 10.1093/eurjhf/hfs105 22828712

[4] Galie N, Humbert M, Vachiery JL, Gibbs S, Lang I, Torbicki A, Simonneau G, Peacock A, Vonk Noordegraaf A, Beghetti M (2016). 2015 ESC/ERS Guidelines for the diagnosis and treatment of pulmonary hypertension: The Joint Task Force for the Diagnosis and Treatment of Pulmonary Hypertension of the European Society of Cardiology (ESC) and the European Respiratory Society (ERS): Endorsed by: Association for European Paediatric and Congenital Cardiology (AEPC), International Society for Heart and Lung Transplantation (ISHLT). Eur Heart J.

[5] Adir Y, Guazzi M, Offer A, Temporelli PL, Cannito A, Ghio S (2017). Pulmonary hemodynamics in heart failure patients with reduced or preserved ejection fraction and pulmonary hypertension: Similarities and disparities. Am Heart J.

[6] Miller WL, Grill DE, Borlaug BA (2013). Clinical features, hemodynamics, and outcomes of pulmonary hypertension due to chronic heart failure with reduced ejection fraction: pulmonary hypertension and heart failure. JACC Heart Fail.

[7] Gerges M, Gerges C, Pistritto AM, Lang MB, Trip P, Jakowitsch J, Binder T, Lang IM (2015). Pulmonary hypertension in heart failure. Epidemiology, right ventricular function, and survival. Am J Respir Crit Care Med.

[8] Vanderpool RR, Saul M, Nouraie M, Gladwin MT, Simon MA (2018). Association between hemodynamic markers of pulmonary hypertension and outcomes in heart failure with preserved ejection fraction. JAMA Cardiol.

[9] Palazzini M, Dardi F, Manes A, Bacchi Reggiani ML, Gotti E, Rinaldi A, Albini A, Monti E, Galie N (2018). Pulmonary hypertension due to left heart disease: analysis of survival according to the haemodynamic classification of the 2015 ESC/ERS guidelines and insights for future changes. Eur J Heart Fail.

[10] Ibe T, Wada H, Sakakura K, Ikeda N, Yamada Y, Sugawara Y, Mitsuhashi T, Ako J, Fujita H, Momomura S (2016). Pulmonary hypertension due to left heart disease: the prognostic implications of diastolic pulmonary vascular pressure gradient. J Cardiol.

[11] Benza RL, Miller DP, Gomberg-Maitland M, Frantz RP, Foreman AJ, Coffey CS, Frost A, Barst RJ, Badesch DB, Elliott CG (2010). Predicting survival in pulmonary arterial hypertension: insights from the Registry to Evaluate Early and Long-Term Pulmonary Arterial Hypertension Disease Management (REVEAL). Circulation.

[12] Kylhammar D, Kjellstrom B, Hjalmarsson C, Jansson K, Nisell M, Soderberg S, Wikstrom G, Radegran G (2018). A comprehensive risk stratification at early follow-up determines prognosis in pulmonary arterial hypertension. Eur Heart J.

[13] Hoeper MM, Kramer T, Pan Z, Eichstaedt CA, Spiesshoefer J, Benjamin N, Olsson KM, Meyer K, Vizza CD, Vonk-Noordegraaf A (2017). Mortality in pulmonary arterial hypertension: prediction by the 2015 European pulmonary hypertension guidelines risk stratification model. Eur Respir J.

[14] Boucly A, Weatherald J, Savale L, Jais X, Cottin V, Prevot G, Picard F, de Groote P, Jevnikar M, Bergot E (2017). Risk assessment, prognosis and guideline implementation in pulmonary arterial hypertension. Eur Respir J.

[15] Pocock SJ, Ariti CA, McMurray JJ, Maggioni A, Køber L, Squire IB, Swedberg K, Dobson J, Poppe KK, Whalley GA (2013). Predicting survival in heart failure: a risk score based on 39 372 patients from 30 studies. Eur Heart J.

[16] Levy WC, Mozaffarian D, Linker DT, Sutradhar SC, Anker SD, Cropp AB, Anand I, Maggioni A, Burton P, Sullivan MD (2006). The Seattle Heart Failure Model: prediction of survival in heart failure. Circulation.

[17] Agarwal R, Shah SJ, Foreman AJ, Glassner C, Bartolome SD, Safdar Z, Coslet SL, Anderson AS, Gomberg-Maitland M (2012). Risk assessment in pulmonary hypertension associated with heart failure and preserved ejection fraction. J Heart Lung Transplant.

[18] Benza RL, Gomberg-Maitland M, Miller DP, Frost A, Frantz RP, Foreman AJ, Badesch DB, McGoon MD (2012). The REVEAL Registry risk score calculator in patients newly diagnosed with pulmonary arterial hypertension. Chest.

[19] Vachiery JL, Tedford RJ, Rosenkranz S, Palazzini M, Lang I, Guazzi M, Coghlan G, Chazova I, De Marco T (2019). Pulmonary hypertension due to left heart disease. Eur Respir J.

[20] Bloos F, Reinhart K (2005). Venous oximetry. Intensive Care Med.

[21] Holm J, Håkanson E, Vánky F, Svedjeholm R (2011). Mixed venous oxygen saturation predicts short- and long-term outcome after coronary artery bypass grafting surgery: a retrospective cohort analysis. Br J Anaesth.

[22] Onoue T, Iwataki M, Araki M, Akashi J, Kitano T, Nabeshima Y, Hei S, Nagata Y, Hayashi A, Tsuda Y (2020). Novel noninvasive estimation of mixed venous oxygen saturation by echocardiography and expired gas analysis. Am J Physiol Heart Circ Physiol.

[23] Bilchick KC, Wang Y, Cheng A, Curtis JP, Dharmarajan K, Stukenborg GJ, Shadman R, Anand I, Lund LH, Dahlström U (2017). Seattle heart failure and proportional risk models predict benefit from implantable cardioverter-defibrillators. J Am Coll Cardiol.

